# Report of Committee Appointed by the Buffalo Medical Association, on an Equitable and Uniform Compensation for Public Medical Services

**Published:** 1849-12

**Authors:** J. B. Samo

**Affiliations:** Chairman of Committee


*Report of a Committee appointed by the Buffalo Medical Association, on an equitable and uniform compensation for public medical services.*

The following report, which was made, some months since, to the Medical Association of this city, was subsequently, by a vote of the Association, communicated to the county Medical Society, and considered by the latter, at a special meeting held in October last, for that purpose. The society, at the meeting referred to, adopted resolutions embodying the recommendations of the committee, with some alterations in the amounts specified as suitable salaries to be affixed to the several offices named in the report, and instructed the same committee to prepare a paper on the subject, for publication in the city papers. Although the report, in one point of view, relates to matters having purely a local interest; in another aspect, concerning the *status* of the profession, it is not thus restricted, and is doubtless as applicable to many other sections, as to the city of Buffalo, and county of Erie. We therefore give it insertion, reserving remarks on the subjects which it involves, for some other occasion.

### REPORT.

In pursuance of the objects of a resolution passed at the December meeting of the Buffalo Medical Association, a committee was appointed to recommend suitable measures for procuring equitable and uniform compensation for medical offices and appointments in this city and county.

The committee, in presenting the following report, would premise, that the offices and appointments, alluded to in the above resolution, are, as is pretty generally known, the following:—County Physician, Physician to the Jail, Physician to the Work House, Medical services on Coroner's Inquests, Health Physician, and Physicians to Cholera Hospitals, should the latter become necessary. They believe, from some reflection given to the subject, that the measures here presented, if not the best that might be devised, are yet tolerably calculated, if properly sustained and carried out, to fulfil the object of the resolution.

It seems to be generally conceded by our physicians, in regard to the object of the resolution, that a reform is highly desirable. But all effort to effect this reform, so desirable, is discouraged by the assertion that it is impracticable. *Why* it is impracticable, your committee are unable to perceive. 'Tis true ("and pity 'tis, 'tis true") want of harmony in the profession is very detrimental to the success of any measures it may prosecute; but it is submitted that a respectable majority is all that is really necessary to be secured, in order to the successful accomplishment of the object in this case. Your committee cannot doubt but that such a majority will sustain this effort. What else, then, is requisite? It seems plain to your committee, that the views and objects of the profession ought to be freely made known to the public. Misrepresentation may thus be counteracted, its aim frustrated; and for this, and other obvious reasons, they should deem it expedient to lay the matter fairly before the people, through the medium of the newspapers. If it should be seen fit to adopt the suggestion, a committee might be appointed to draw up a plain statement, showing, especially, the true motives of the profession in the matter, and directing attention to these principal points, viz.

*First,* The undeniable necessity that exists for additional appropriations for the poor.

*Second,* An almost equal necessity for a revision, or, it may be, reconstruction of the medical department of the Alms House.

*Third,* The stand taken by the profession upon the subject of compensation.

It may be thought your committee are transcending the limits of their duty, in some of these suggestions. But, a little reflection, they think, must satisfy every unprejudiced medical man of the close relation they bear to the object of the resolution.

*First,* In regard to to the urgent, existing necessity for additional appropriations for the poor. That this subject should engage the attention, and enlist the efforts of medical men, both in regard to their professional character, and their character as christians, your committee think none here can deny; and also, that it is equally the province of our profession to explain and elucidate it to the public. It has been already most ably discussed by the editor of the Buffalo Medical Journal, and in a manner that should elicit a warm approval from the humane and intelligent everywhere. It may be asked by some, in what way appropriations for the poor are connected with the object of the resolution. In reply, your committee would ask, whether, with the present inadequate salaries of physicians employed by the county, it be not fair to infer that the sick under their charge, must, in too many instances, forego the advantages derivable from the best known methods of treatment, simply because it cannot be afforded at the present low rates of compensation. (so miscalled). They appeal to any gentleman who has held a county medical appointment, whether this be not, to a certain extent, true. But these need no arguments to prove the correctness of this inference to the profession. It may not be so readily entertained by the community, however, and your committee would suggest the propriety of exhibiting some illustration of the fact. As one of the most familiar, of daily recurrence, and of striking importance, take the following:—It is well known to the profession that we possess few remedies having more valuable qualities than quinine, and also that few diseases, comparatively speaking, occur, in which, at some period or other, its employment is not highly useful. In some diseases it seems almost indispensable—a *sine qua non.* Are your committee too bold in hazarding the assertion, that this medicine is not commonly employed in county practice, solely on account of its cost; and in most cases demanding its use, a substitute for it, of far less value in every sense of the term, is employed? Another instance of equal, it may be said, greater importance, as a remedy, is the hydriodate of potassa. Your committee regret they cannot speak more positively in this place, and would request the incumbents of county medical offices to testify on this point; but they think little doubt need be entertained, even by the most charitably disposed, as to the infrequency of its employment. How disadvantageous to the patient, any attempt at a substitute for it must be, medical men alone are aware. So also of other preparations of iodine; so, to a greater or less extent, it is presumed, with the nitrate of silver, the preparations of morphia, and some others, medicines of the first rank as remedial agents, whose employment, in private practice, is deemed indispensable, but which, from their greater cost, are, to a considerable extent, doubtlessly, replaced by others of less cost, and far less remedial value. This is a fair sample of the mischiefs that spring from the present arrangement, and the fact should be generally made known. In the language of the article already alluded to, "is this a matter of no interest or importance in the minds of the citizens of Erie county?" Is it a matter in which no moral responsibility is involved?

*Second*, As respects the necessity existing for a revision or reconstruction of the medical department of the alms house, your committee are not prepared to go into any details, or even to give the requisite outline of what it should be. They desire, however, upon this occasion, to protest against one feature of the present system, making it incumbent on the physician to furnish medicines for those under his care. They cannot but view it as fraught with evils, and not only injurious to his usefulness as a physician, but derogatory to his professional character, and detrimental, without a doubt, to the interests of his patients. They would urge, therefore, by considerations of interest, as well as humanity, that the physician be confined to his legitimate duties. Your committee, in this place, though perhaps not fairly belonging to it, cannot refrain from alluding to the pernicious practice of holding up medical offices to the lowest bidder, just as proposals are invited for paving a street, or building a sewer. Can any thing be better calculated to degrade the profession, and pander to a mercenary and unprincipled spirit, too ready at all times, to lift its head among us? In the language of the President of the State Medical Society, in his address recently delivered in the Capitol, at Albany, "We claim to constitute, or represent, a liberal profession: and the very idea or essence of a liberal profession, as distinguished from a trade, is, that the acquisition of money is not its primary object. Nor is it so with physicians." Yet do we not tacitly admit that we are pursuing a trade, when, to quote the language of the physicians of Little Falls on a recent occasion, "we allow our professional services to be made a matter of merchandize, and the subject of pecuniary competition." Your committee would further suggest, in relation to this part of their subject, that the new arrangements recently adopted in the medical department of the Bellevue Hospital, in the city of New York, and highly spoken of, and approved by medical men, be examined into, with a view to their adoption here, if found satisfactory and applicable to this county. The observations in the foregoing relating more particularly to the alms house, will, nevertheless, apply, to a greater or less extent, to the jail, and work house.

With regard to a minimum compensation, one that shall be equitable and uniform, your committee have found it difficult to arrive at very definite conclusions. They have sought, though vainly, for some statistics to show the average number of sick annually in charge of the county physician, both in and out of the alms house. They might have thus approximated to the amount of services performed, sufficiently near to have determined with some precision, an equitable minimum compensation. But they could find no records either with superintendent, overseers, or physician. This may, or may not be essential; it has proved somewhat inconvenient to your committee in their search for data. They would say, however, that by three of the most respectable physicians of this county, who have held the appointment, $1000 has been stated as the lowest compensation for which it its duties can be faithfully and efficiently performed. If this be a correct estimate now, it cannot be believed that a less amount will be required in future. On the contrary, are not the probabilities strong, that, ere long, the present number of poor will be greatly augmented? If your committee venture to judge by those "signs of the times" exhibited in the "old world," they should anticipate a great influx of the poor and destitute thence, and a consequent increase of the business of the county physician.

With regard to the Jail appointment, your committee can only give, as in the case of the alms house, the estimate of gentlemen who have held it. The majority incline to place the minimum at $100. While some have fixed it higher, and others lower, $100 is doubtless low enough, but but may be considered equitable, if the requirements now existing in relation to the furnlshing of medicines be expunged. Your committee would again protest against this conjunction of the duties of the apothecary with the already sufficiently arduous and harrassing duties of the physician. They earnestly hope, ere long, to see it altogether reformed.

The appointment of physician to the work house being comparatively of recent date, there are not, of course, as in the two other county medical appointments, the same facilities for making up an estimate of a just minimum compenaation. From all your committee have learned, however, in relation to it, they feel but little doubt that the amount of medical services is greater, and more onerous at this establishment than at the Jail, which, added to its being in a far less accessible locality than the latter, induce them to place the minimum at $200, This, however, is the more open to discussion, as your committee acknowledge they have not given it the investigation its importance demands.

Services on Coroners' Inquests come next to be considered. The nature and amount of these are various. In fixing a minimum, therefore, your committee, guided by the opinion of respectable medical men, whose experience in the matter has been greatest, have fixed upon two rates of compensation; the same, it is believed, at present allowed by the county. For making post mortem examination, $5.00. For viewing a body, testifying before a coroner's jury, &c., $3.00. Your committee would suggest, in addition to this, that as in making post mortems, it is often necessary that two or more engage in it, each and every physician so engaged shall be entitled to, and receive $5.00. It is believed that heretofore, or, at any rate, of late, the County has refused to compensate more than one in each particular case. This, it would seem, demands reform.

The appointment of Health Physician comes next in order. It seems to your committee, however, to call for no suggestions from them in relation to the compensation attached to it.

The subject of Cholera Hospitals completes the list. If it be considered upon how many circumstances and accidents the value of medical services in these establishments must depend, it will be perceived how vague must be the estimate of what would be a just minimum compensation. But yet, to your committee, it seems very desirable, as of great importance, that this part of their subject be not left indeterminate. To all who have reflected upon the aim and scope of the resolution, this must be seen to farm an important part of it. Your committee have attempted, in their report, to show some of the evils, moral as well as physical, attendant upon, if not resulting from inadequate compensation in County medical appointments. Is it not apparent that this also would be liable to the same abuses we have considered in the case of the others? Your committee would therefore urge, as a duty to ourselves, and the public, that the subject of Cholera Hospitals be discussed, and something definite in regard to compensation fixed upon. From all they have gathered on the subject, from those physicians of our city deemed best informed upon it, your committee would incline to submit $5 per day as an extreme minimum compensation. This, however, is rather suggested for the sake of eliciting the views of others, than from any opinion of its propriety.

In conclusion, your committee must beg the indulgence of the association for the feeble and imperfect manner in which they have discharged the duty assigned them. The subject, all must admit, is not very inviting in its aspect. Reform in these days has come to be considered as nearly synonymous with humbug. Attempts at innovation, also, are deservedly eyed with suspicion; they are but too often the offspring of the visionary, or it may be of restless aspirants, whose position under the established order of things is by by no means suited to their ambitious views, or accordant with the notions entertained by them of their own importance. Your committee trust they are not obnoxious to suspicions of this nature. It could not be expected of their report, that it should embrace and obviate all the possible difficulties with which the subject of it is involved. It could not be expected to reconcile those unfortunate differences in the profession that must ever stand as a barrier in the way of reform. Would that it could! Would it were in the power of your committee to inspire such a spirit of concession and conciliation among us as would prompt the casting aside of all animosities, and our blindly selfish interests, a spirit that would gently lead us, as it were, to some moral eminence, whence, with a comprehensive glance, we might survey the whole of life, with its true ends and aim. Your committee are ready to acknowledge they may have misapprehended their subject; or not have given an accurate exposition of it. They may have taken too narrow or too extended views for practical purposes; whatever judgment the Association may pass upon them, they must still claim for their efforts the humble merit of rectitude of intention.

All of which is respectfully submitted. J. B. SAMO,

*Chairman of Committee.*

